# Chiropteran types I and II interferon genes inferred from genome sequencing traces by a statistical gene-family assembler

**DOI:** 10.1186/1471-2164-11-444

**Published:** 2010-07-21

**Authors:** Thomas B Kepler, Christopher Sample, Kathryn Hudak, Jeffrey Roach, Albert Haines, Allyson Walsh, Elizabeth A Ramsburg

**Affiliations:** 1 Center for Computational Immunology, Duke University Medical Center, Durham, NC, USA; 2 Duke Human Vaccine Institute, Duke University Medical Center, Durham, NC, USA; 3 Lubee Bat Conservancy and Department of Wildlife Ecology and Conservation, University of Florida, Gainesville, FL, USA

## Abstract

**Background:**

The rate of emergence of human pathogens is steadily increasing; most of these novel agents originate in wildlife. Bats, remarkably, are the natural reservoirs of many of the most pathogenic viruses in humans. There are two bat genome projects currently underway, a circumstance that promises to speed the discovery host factors important in the coevolution of bats with their viruses. These genomes, however, are not yet assembled and one of them will provide only low coverage, making the inference of most genes of immunological interest error-prone. Many more wildlife genome projects are underway and intend to provide only shallow coverage.

**Results:**

We have developed a statistical method for the assembly of gene families from partial genomes. The method takes full advantage of the quality scores generated by base-calling software, incorporating them into a complete probabilistic error model, to overcome the limitation inherent in the inference of gene family members from partial sequence information. We validated the method by inferring the human IFNA genes from the genome trace archives, and used it to infer 61 type-I interferon genes, and single type-II interferon genes in the bats *Pteropus vampyrus *and *Myotis lucifugus*. We confirmed our inferences by direct cloning and sequencing of IFNA, IFNB, IFND, and IFNK in *P. vampyrus*, and by demonstrating transcription of some of the inferred genes by known interferon-inducing stimuli.

**Conclusion:**

The statistical trace assembler described here provides a reliable method for extracting information from the many available and forthcoming partial or shallow genome sequencing projects, thereby facilitating the study of a wider variety of organisms with ecological and biomedical significance to humans than would otherwise be possible.

## Background

Novel human pathogens appear at a continually increasing rate. The majority of these agents are zoonotic, and have their origins in wildlife [1]. Among wildlife sources, bats, mammals of the order *Chiroptera*, are perhaps the most striking, serving as the primary natural reservoirs for many human pathogens [2-5], including several viruses of notorious lethality in humans: Nipah virus [6], Hendra virus [7], Ebola and Marburg Viruses [8,9], and Rabies virus. Rabies virus is regarded as the most lethal of all human pathogens, and has been found in bats for over a century [10]. Rabies and other Lyssaviruses are now found in many other animals, but phylogenetic studies indicate that these viruses first evolved in bats and transferred to animals of the order *Carnivora *more recently [11]. SARS-like Coronaviruses have also been found in bats [12,13] and so far provide the closest link to the agent of human SARS.

Infection by each of these viruses in bats appears to be asymptomatic or of greatly reduced pathology. Even Rabies virus, for which we have a great wealth of experimental data, and which is regarded as invariably lethal in other mammals, has been shown repeatedly to have decreased virulence in bats [14].

The association of so many human-lethal viruses with bats may be partially attributable to the fact that the order *Chiroptera *to which the bats belong, is the second largest mammalian order, behind only *Rodentia *in total species [15]. On the other hand, the unique lifestyles and ecological circumstances of bats makes plausible the idea that the coevolution of bats and their viruses produced unusually virulent viruses and a host antiviral response tuned to suppress them.

If the latter is the case, it will be of great interest to understand the ways in which the several components of the bat antiviral response differ from those in humans and other mammals. The main hurdle to the pursuit of this goal is our paucity of knowledge of bat immunology and almost complete lack of reagents to initiate such studies. What we do have now that was not available a few years ago is the information obtained through large-scale sequencing projects. This information resides, for the most part, in partially-or unassembled genome sequences, and is not practically available for such purposes without further technical developments.

Our aims in this paper are to describe the method we have devised for the partial assembly of genome sequencing traces for the inference of gene families, to validate this method on the human interferon alpha family, and to use it for the inference of the type-I interferon families in the bats *Myotis lucifugus *(little brown bat) and *Pteropus vampyrus *(large flying fox).

Most pathogenic viruses, including all of the viruses listed above, possess one or more genes that directly antagonize the type-I interferon response [16-19], indicating a strong coevolutionary pressure between these viruses and the type-I interferon system. In experimental infections of mice with Rabies virus, the investigators noted a marked correlation between survival and IFN production [20].

Type I inteferons are the primary mediators of one of the earliest stages of the antiviral response in mammals. They are induced rapidly and secreted upon viral infection. Cells exposed to these cytokines enter an infection-refractory state induced by signaling through the common type-I interferon receptor complex. In this state, transcription and translation of viral gene products is inhibited, MHC class I expression is upregulated, RNA and protein degradation are accelerated (reviewed in depth in refs [21-23]). There are 13 human functional IFNα genes, as well as single functional genes for IFNβ, IFNε, IFNω, and IFNκ The type 1 interferons are highly pleiomorphic, exhibiting both distinct and complex overlapping function [21,24]. All of the known mammalian type-I interferons genes are intronless; their transcripts are neither spliced nor edited before translation, and expression is controlled at the transcriptional level [23,25,26].

### Bat Genome Projects

There are two bat whole-genome sequencing projects in progress, but neither is complete at this point. One of them will provide coverage at a level that will not produce a reliable assembly. There are in fact a great many sequencing projects underway for which there is no intent to do full coverage [27]. The *Myotis lucifugus *genome sequencing project has completed sequencing to approximately 7-fold coverage with 27,486,306 traces thus far. The genome is, as of this writing, being assembled http://www.broadinstitute.org/science/projects/mammals-models/brown-bat/little-brown-bat. The *Pteropus vampyrus *genome sequencing project has produced 8,051,001 genome traces http://www.hgsc.bcm.tmc.edu/project-species-m-Megabat.hgsc?pageLocation=Megabat and is slated for completion with 2x coverage. The *Pteropus *project is based on samples from a single individual bat (personal communication).

The consequence of incomplete coverage is the exacerbation of one of the shortcomings of whole-genome shotgun sequencing: the difficulty of resolving repetitive DNA segments [28]. If two sequencing traces contain regions of similarity, it may be difficult or impossible to determine whether these traces are derived from the same underlying DNA or from two distinct DNA segments that are themselves paralogous. This difficulty is not limited to the assembly of highly repetitious intergenic regions, but to the inference of gene families as well.

This latter problem is particularly unfortunate, because gene duplication is a major source of innovative potential in evolution, and the comparative study of gene families among related species is therefore of great interest [29]. Furthermore, a large proportion of genes in eukaryotic genomes reside in families, including the genes that encode the type-I interferons.

In this paper, we describe a method for the inference of gene family members from unassembled sequencing traces. The method is conceptually straightforward, and is based on an information-theoretic model that accounts for both sequencing error and evolutionary divergence, providing the means to encode the set of sequencing traces. We then seek those partial assemblies that make the total description length of the combined set of sequencing traces as small as possible. This reconstruction provides an estimate of the number of genes in the family and posterior probability mass functions on the DNA sequences of these genes.

We first present the model and the algorithm we have developed to minimize the description length and thereby infer the structure of the gene family. We validate the methods by reconstructing the human type-I interferon family genes from sequencing traces from the human genome project. We use this method to infer the type-I interferon families from the sequencing traces from the *Myotis lucifugus *and *Pteropus vampyrus *genome sequencing projects. We examine these genes in comparison with the orthologous families in humans and other mammals. Finally, we confirm our inferences by cloning and sequencing genes from four of the interferon families.

## Results

### Inference of Gene Families From Trace Archives

In this section, we present the mathematical basis of the assembly method we have developed and provide a concise algorithmic implementation.

We assume a set *S *of sequencing traces containing regions with similarity to some given gene or genes. In practice, this seed gene would be an ortholog of the genes one is attempting to infer; one collects *S *using similarity searching on the complete trace archive for the species of interest.

**Figure 1:**
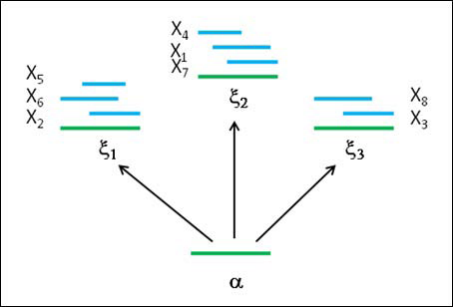
**Schematic of the statistical model**. The observed sequencing traces *X*_i _result from the observation of genes ξ. These genes are related by descent from the common ancestor α.

There are *g *genes in the family, where *g *is an unknown integer. Each of these genes is related to a common ancestor α (whose sequence is also unknown) and differs from α by the accumulation of mutations, including insertions and deletions. Each sequencing trace results from direct templating from one of these genes, and differs from it by sequencing error only. Figure 1 illustrates the statistical model used for the inferential procedure.

Given any two sequencing traces in *S*, they are related either through sharing the same immediate template (traces 5 and 6 in figure 1, for example), or indirectly, through the ancestor, α, with each having a distinct immediate template. These two alternatives correspond to two different likelihoods. In the first, the differences between the two traces are attributable to sequencing error only, and must be consistent with the reported position-specific quality scores. In the latter, the divergence of the underlying genes from their common ancestor provides an additional source of differences. The difference in description lengths under these two models provides a criterion for choosing between the models. In fact, the criterion we will use for determining the final set of assemblies is the minimization of the total description length of *S *under the model in figure 1. Briefly, the description length is the information required to encode the data and the values of the model parameters. (See [30] for a complete treatment of description length techniques for inference.) For any sub-model for pairs of traces that involves mutations from the common ancestor, the description length must account for the information required to encode the mutation rate. If *y *is the overlap in nucleotides between the two sequencing traces, the cost of encoding the mutation rate is log *y*.

The objective of the assembly procedure is the minimization of the description length over all topologies of the form shown in figure 1, but there is no method for finding this minimum, short of exhaustive enumeration over all topologies, which is ruled out by practical considerations for all but the smallest *S*. We therefore adopt a greedy progressive method familiar from its use in multiple sequence alignment. At each stage, we find the pair of sequences (traces or partial assemblies) that provide the maximum saving in description length and assemble them, replacing the pair by the new assembly they have formed. The process continues until there are no remaining moves that lead to a description length reduction.

The following section provides the details for the probability model.

#### Quality scores and conditional probabilities

The base-calling software reads the raw sequencing trace and, for each position, reports a single base and a quality score. If the reported base is *X*, and the reported quality score is *q*, we take the output to represent a posterior probability mass function (pmf) on the latent nucleotide state at that position, given by(1)

where *D *represents the data conditioning the estimate, and ε(*q*) is defined by(2)

It is not necessary to specify a likelihood function, or even to describe what the nature of *D *is, so we simply write for the posterior probability mass function on ξ in the *j*th position in the *i*th sequencing trace. For simplicity in this presentation, we will assume that the prior pmf on ξ is uniform on A, C, G, T, -.

We will also need the posterior pmf on the ancestral state α, which is obtained by specifying a model for the accumulation of mutations from α to ξ, and summing over the unobserved state, ξ. We take a simple reversible mutation model(3)

and let(4)

Consider the model partially described by having position *i *in trace 1 aligned with position *j *in trace 2. The posterior pmf for the latent true nucleotide state is(5)

where *k *is the position in the assembly corresponding to *i *and *j *in traces 1 and 2, respectively. is given by(6)

Since the criterion is the minimization of the description length we compare any two models by the difference in their description lengths. The alignment cost, then, is the difference in description length between the model in which these positions are aligned (given the alignment) and the one in which they are independent, and is give by(7)

Denote by ***A***(*k*) the vector whose components are the indices for the aligned traces corresponding to the *k*th position in the resulting assembly. Then the cost of the alignment is(8)

For the case where traces 1 and 2 are related indirectly through a common ancestor, we similarly have(9)

with the appropriate and obvious definitions. The quantity of primary importance for us is the cost difference between the direct and indirect models for a given pair of traces(10)

The logarithmic term is the cost of encoding the mutation rate that minimizes the alignment score [30]; the argument  is the number of nucleotides in the overlap of traces 1 and 2 in the optimal alignment, ie, the number of degrees of freedom available to estimate μ. When *c *is positive, the indirect model is the more efficient; when *c *is negative, the direct model is more efficient.

**Figure 2:**
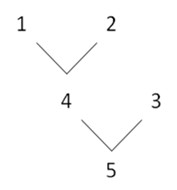
**Diagram indicating the labeling of traces and assemblies for the discussion of progressive alignment in the text**.

For progressive alignment, note that the posterior assembly pmf and the cost are composed from the individual sequential alignments. Suppose we first align traces 1 and 2 to obtain assembly 4, and then align trace 3 to assembly 4 to produce assembly 5 as shown in figure 2. We need to refine our notation for indexing by letting refer to the position in trace *i *corresponding to position *k *in assembly *a*. So, for the example of figure 2, we have(11)

It is then straightforward to show that the alignment scores are additive:(12)

and that the assembly posterior pmfs are obtained by composition(13)

We have shown that the total cost for the composite alignment is given by the sum of the sum of alignment costs, but, as with all progressive alignment methods, the minimum-cost composite alignment may not be the global minimum cost alignment.

The complete procedure, then, is

1. Compute all of the minimum pairwise alignment costs *c_ij _*using dynamic programming.

2. Assemble the two sub-assemblies with least alignment cost, and replace them with the new assembly that results. Compute the pairwise costs between the remaining subassemblies and the new assembly.

3. Continue at step 2 until total assembly cost can no longer be reduced.

### Validation

We validated the method by inferring the human interferon-alpha (IFNA) gene family directly from the human whole-genome sequencing trace archives, and comparing the results to IFNA gene sequences known from the finished human genome (as well as from decades of molecular biology on the human IFNA). We performed this procedure in three different ways.

First, we collected relevant sequencing traces by performing a BLAST search using the 567 nt-long protein-coding portion of the human IFNA2 transcript (NM_000605) and retrieved 291 traces with expected values less than 10^-6^. We trimmed each sequence and applied the assembly method described above.

The procedure resulted in 15 final assemblies. We compared the inferred genes to the human genome reference assembly. Each of these 15 assemblies maps unambiguously to one of the 13 known IFNA genes or the single whole pseudogene at an average error rate of 6.0 × 10^-4 ^per base (14 mismatches in 23374 bases). In one case, a pair of assemblies mapped to a single genomic gene, IFNA17. There are 2 single nucleotide differences between the two assemblies mapping to IFNA17 and one dinucleotide difference. Each of the single nucleotide differences is strongly supported, with multiple high-quality reads supporting the inference. The dinucleotide difference is less well supported, with just a single trace covering it in one assembly, though the corresponding bases in this trace are good quality. The evidence strongly suggests that the two assemblies do map to two different alleles of the same gene. Accepting this conclusion reduces the error rate to less than 4 × 10^-4 ^per base. The 10 remaining mismatches include a single 4-nucleotide deletion relative to the genomic sequence.

For the next two validation procedures, our intent was to more accurately represent the situation in which the assembler would be used. For this purpose, we used one of the *P.vampyrus *putative IFNA genes rather than human IFNA2 as the BLAST query, and selected a subsample of the retrieved sequences to simulate low coverage.

There were 74 trace reads in the subset produced by the Whitehead Institute. Assembling these reads using our method produced 13 distinct assemblies with two or more reads in each, mapping (as described above) to 12 different human genomic regions: 7 IFNAs, human IFNW1, LOC100130866 (annotated by NCBI as similar to IFNW1), 2 IFNA pseudogenes and 1 IFNW pseudogene. One pair of distinct assemblies was mapped, erroneously, to a single IFNA. The subset of trace reads from the Venter Institute consisted of 103 sequences, producing 22 distinct assemblies with 2 or more reads, mapping to 22 different regions of the human genome: all 13 IFNAs, 1 IFNA pseudogene, IFNW1, and 6 regions annotated as either IFNW-like or IFNW pseudogenes. The alignments of these assemblies to the genome revealed 3 mismatches in 31531 total bases. One pair of assemblies was erroneously mapped to a single IFNA pseudogene.

### Type I interferons in *Myotis lucifugus *and *Pteropus vampyrus*

We used the protein-coding portions of the human genes IFNA2, IFNK, IFNB, and IFNE, the bovine gene IFNT, and murine gene IFNZ in blast searches against the whole genome shotgun sequence trace archives for *Pteropus vampyrus *and *Myotis lucifugus*. The sequences retrieved in each of the blast searches were then assembled using the methods described above.

We obtained unique final assemblies for IFNB in each bat. Each of these final assemblies contained an intact ORF which, when BLASTed against the NCBI nucleotide sequence database, returned multiple hits on the corresponding gene in several mammals. The greatest similarity was to horse, pig, and cow sequences, with about 80% nucleotide identity.

*Myotis *produced two final assemblies for IFNE, both of which contain intact ORFs and appear, on close examination, to be genuinely distinct sequences. *Pteropus *produced a single final assembly, which had an intact ORF.

The sequencing traces returned in the search on IFNA2 produced multiple final assemblies that fall into three distinct families with clear similarity to IFNA, IFNW, and IFND (Table 1), respectively.

**Table 1:** Summary of Final Assemblies in *Myotis lucifugus *and *Pteropus vampyrus*.

Species	Family	#assemblies	#Intact ORFs	#Pseudogenes	#Partial	length (AA)
*M. lucifugus*	IFNB	1	1	0	0	186
	IFNE	2	2	0	0	193
	IFNK	2	2	0	0	208
	IFNA	2	0	2	0	-
	IFNW	25	12	7	9	195
	IFND	19	11	3	5	171^1^

*P. vampyrus*	IFNB	1	1	0	0	185
	IFNE	1	1	0	0	193
	IFNK	1	1	0	0	201
	IFNA	7	7	0	0	189
	IFNW	28	18	8	1	185^2^
	IFND	14	5	7	2	171^3^

1. two genes encode polypeptides of length 190

2. one at 187 and two at 195

3. one at 170

No relevant hits to murine IFNZ were found in either chiropteran genome. No hits to bovine IFNT beyond those already obtained using other queries were found.

#### Chiropteran Type II interferon

In mammals, the type I interferons have a single exon of fewer than 700 bases. IFNG, the type II interferon, has multiple exons. We used our methods to infer the type-II interferon genes in the two bats by using the full-length human IFNG gene comprising introns and exons (4972 bases, NC_000012 REGION: complement(68548550..68553521)). Our methods produced a single assembly in each bat (Additional File 1). All exons were clearly identifiable and all intronic splice signals were conserved. Table 2 shows the sequence similarity among the human and bat sequences.

**Table 2:** DNA sequence similarity among human (Has) IFNG and inferred bat (Mlu, Pva) IFNG assemblies for exons (above the diagonal), and introns (below the diagonal).

species	Hsa	Mlu	Pva
Has	ID	0.752	0.784
Mlu	0.655	ID	0.832
Pva	0.711	0.696	ID

### Phylogenetics

**Figure 3:**
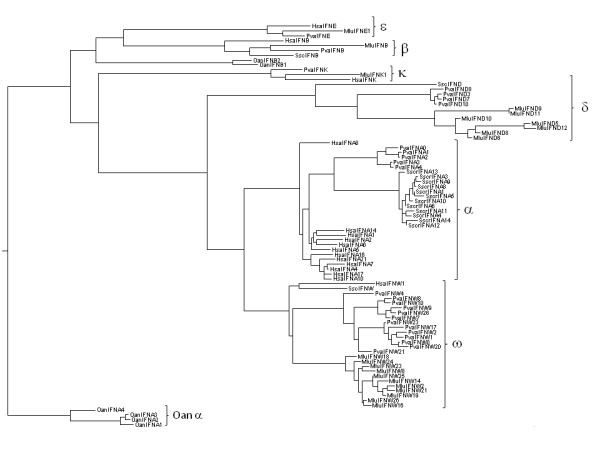
**Phylogenetic tree of the type-I interferons in *Pteropus vampyrus *(Pva), *Myotis lucifigus *(Mlu), *Sus scrofa *(Ssc), *Homo sapiens *(Hsa), and *Ornithorhynchus anatinus *(Oan)**. The tree was thinned for clarity by omitting one member of any pair of sequences differing by fewer than 3 amino acids. The branch length is proportional to the evolutionary distance.

The intact ORFs of the inferred sequences were aligned with the type-I interferons from humans and from the domestic pig, *Sus scrofa *(which is substantially more closely related to bats than is the human), and as an outgroup, the IFNB and IFNA gene sequences from the duckbill platypus (*Ornithorhynchus anatinus*). The phylogenetic tree relating these genes was inferred using maximum likelihood (Figure 3).

The features of the inferred phylogenetic tree are consistent with those found in previous investigations [31]. In particular, the interferon families fall into distinct clades, with the exception of the platypus IFNA genes, which appear ancestral to the IFNA, IFNW, and IFND genes of the other species. Within each of the families, the genes fall into clades according to species, with no interdigitation of genes from different species in any clade (Figure 3). Additionally, the bat genes from one bat species are typically, but not universally, more closely related to the orthologous genes from the other bat species than to those of pigs or humans. The bat genes are typically but not universally more closely related to the orthologous pig genes than to the orthologous human genes.

### Sequencing

We used the inferred sequences to design sequencing primers and make several clones from each of IFNA, IFNB, IFND, and IFNK. The results are as follows.

We obtained DNA sequences from 19 independent IFNB clones from two individual bats. Among these, 2 distinct but very closely related genes were clearly discernible. One sequence is identical to the consensus of all 19 sequences, and is found in 7 of 12 clones from one bat, and all the clones from the other. Given this interpretation, the overall frequency of pre-sequencing PCR error was 1.6 × 10^-3 ^per base (17 errors in 10792 bases) The inferred IFNB genes are 91% identical to an IFNB isolated from *Rousettus aegyptiacus *[4] and 80% identical to IFNB from the domestic pig, *Sus scrofa*.

Using IFNK primers, we obtained sequences from 7 clones from a single animal, and found two distinct genes, differing from each other in two nucleotides. The error rate was consistent with that found in the IFNB genes at 1.7 × 10^-3 ^per base (6 errors in 3430 bases).

For IFND and IFNA, respectively, we collected sequences from 32 and 52 clones from a single animal. Because PCR was used in an amplification step prior to cloning (unlike the process used for genomic sequencing), it is difficult to discern the expected genetic variability from sequencing error. For IFNA, four sequences were recovered multiple times from independent procedures. The mean number of mismatches in pairwise comparisons is just under 7. The four sequences are within 1, 2, 5 and 6 bases of the most similar inferred IFNA gene. For IFND, there are three multiply-represented sequences. Two of these genes differ at 9 bases; the third, which represents a pseudogene, differs from the other two by more than 20 bases. These genes differ from their nearest inferred IFND sequences by 7, 12, and 20 bases, respectively. Additional file 2 contains an alignment of the three multiply-represented IFND clones compared to the IFND assemblies.

**Figure 4:**
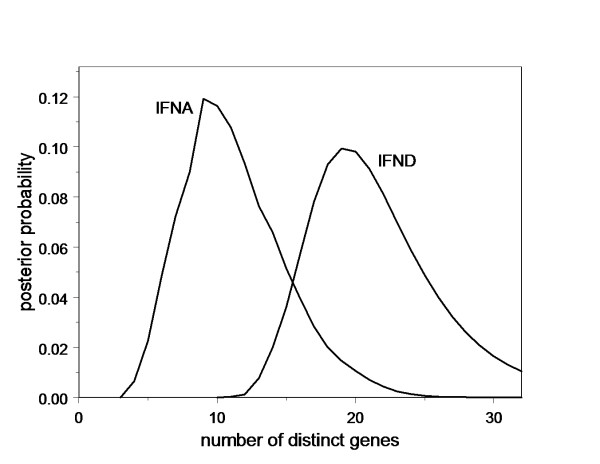
**The posterior probability mass function on the number of genes in the IFNA and IFND families as estimated by cloning and sequencing from peripheral blood cells from *Pteropus vampyrus***.

Since it was not possible to determine by inspection what the underlying true gene sequences are or how many there are. We instead estimated the posterior probability on the number of genes in each family as described in Methods (Figure 4). For IFNA, the posterior probability is maximized at g = 9 genes; the 90% credible interval is (6,17). For IFND, the greatest posterior probability occurs at g = 19 genes, and the 90% credible interval is (15,28).

### Gene expression

To test whether the inferred genes are expressed under conditions known to induce type

I interferons, we designed primers to *Pteropus *IFNB and to a control housekeeping gene (PPIA) and a gene (OAS2) induced by paracrine IFNB signaling. The latter two genes were also inferred by the methods described.

Fresh *Pteropus *PBMCs from two individual animals were cultured under four different conditions: with lipopolysaccharide (LPS), with the synthetic RNA poly(I:C), with Vesicular Stomatitis Virus (VSV), and with culture medium alone.

**Figure 5:**
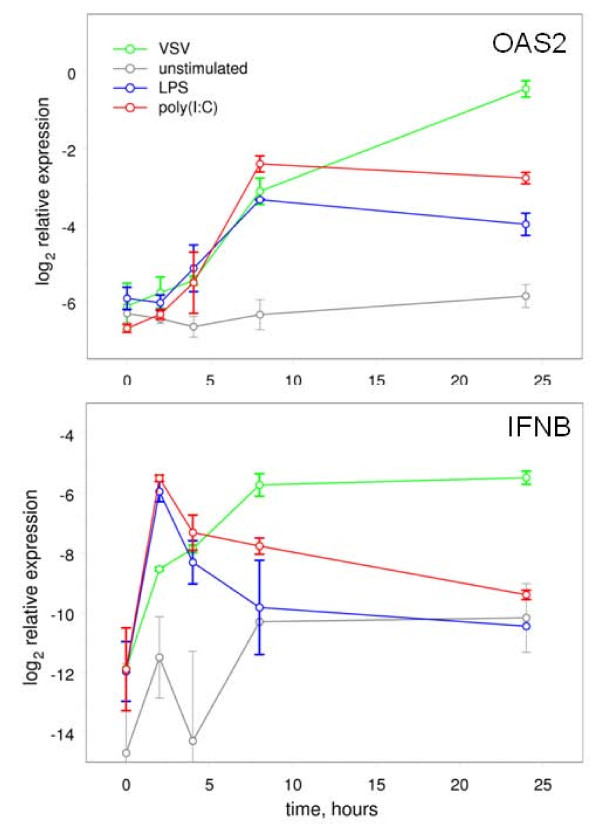
**Gene expression time course by qRT-PCR in fresh *P.vampyrus *PBMCs stimulated in culture by the indicated compound**. Expression levels are relative to the housekeeping gene PPIA. The error bars represent standard errors of the mean over biological duplicates.

With LPS or poly(I:C) treatment, IFNB mRNA expression was increased 20-50 fold by 2 hours and decreased slowly to near the starting value by 24 hours. OAS expression lagged behind that of IFNB, reaching its peak value by 8 hours, declining from the peak level only partially by 24 hours. In contrast, cells treated with VSV had peak levels of IFNB appear by 8 hours and remain almost unchanged to 24 hours; OAS expression rose more slowly, and had not reached a maximum level by 24 hours (Figure 5).

## Discussion

Recent research has demonstrated very clearly that the rate of emergence of human pathogens continues to increasing steadily, and that the source of the majority of these agents is wildlife [1]. One of the most intriguing findings of late is that bats are the natural reservoirs of many of the most pathogenic viruses in humans.

While it is known that microbe-host coevolution drives pathogenicity in the natural host, the effect of such coevolution on alternative hosts has not been described. The development of many genome sequencing projects extending beyond domesticated animals provides an opportunity to begin such inquiries. The low coverage levels of these projects and the fact that so many genes with immunological functions appear in large families of very similar genes requires the development of more precise inferential tools for their study.

Toward that end, we have developed a method for the assembly of genome sequence fragments for use in the inference of gene family members when the genome coverage is too low for reliable complete assembly. We validated the method on raw, unassembled traces from the human genome sequencing project, finding that we could assemble the known interferon alpha sequences accurately, and further, identify at least one case in which two alleles are present.

It should be noted that our method requires more computation than assembly methods currently in use due to the numerical minimization over the mutation frequency required in Eq(10), and is therefore not suitable for large-scale assembly.

Using this method, we inferred a total of 61 type-I interferon genes with intact ORFs from the whole-genome shotgun trace archives for two chiropteran species, *Pteropus vampyrus *and *Myotis lucifugus*. We find that the largest of the IFN-I gene families in both bats comprises genes orthologous to the IFNW genes in other mammals. In humans, mice and pigs, there is just one IFNW gene but up to two dozen members in each bat. A recent analysis of bovine type-I interferons from the assembled *Bos taurus *genome [32] finds 26 intact IFNW genes, providing precedent for our otherwise striking results. In contrast, the IFNA family is the largest IFN-I family in several mammals, including humans, mice and pigs, but appears to be smaller in *Pteropus *and absent but for pseudogenes in *Myotis*. The gene family assembly from trace archives indicates that there are 7 intact IFNA in *Pteropus*; analysis of direct sequencing from PBMCs gives maximum posterior probability to the presence of 9 IFNA genes (Figure 4), with a 90% credible interval containing 6 to 16 genes. Cattle have 13 IFNA genes in spite of having a greatly enlarged IFNW family [32].

Both bats have multiple members in the IFND family with five intact members and seven pseudogenes in *Pteropus*, and twelve intact members in *Myotis*. IFND has been found as a functional gene only in pigs, where the gene product is expressed in the placenta and plays a role in embryonic development [33], but is not suspected of involvement in the response to viruses. It is striking to us that this family seems to have been so dramatically enlarged in bats. The size of this family suggests that it may still be involved in host defense in bats even if it has lost that function in pigs. Walker and Roberts [32] report finding three IFND pseudogenes, but no intact IFND in the cow. It is worth noting that the placental type-I interferon in cattle is IFNT rather than IFND [34]. Searching the bat trace archives with bovine IFNT did not produce hits that had not been returned with the other searches. We find no evidence of IFNT or IFNZ in either bat.

For IFNB, IFNK, and IFNE, we find one member of each in *P. vampyrus*, and one or two in *M. lucifugus*. In the case where we do find two genes, we are confident that there the genes are distinct, though they may represent alleles rather than paralogs.

We used the inferred sequences for *P. vampyrus *IFNA, IFNB, IFND, and IFNK to design oligonucleotide primers for cloning and sequencing and recovered a total of 110 sequences. The directly cloned sequences validate much of the gene family inferences; most of the repeated sequences are within a few bases of the nearest inferred gene. A minority of the directly sequenced genes are surprisingly far from the nearest inferred gene. This circumstance may be an indication that there are additional type-I interferon genes not covered by the Pteropus sequencing traces, and may also reflect significant population polymorphism in the wild bat population.

We used these same primers to show, using quantitative RT-PRC, that the *P. vampyrus *candidate IFNB and OAS2 (a gene induced by IFNB in other mammals), are expressed upon stimulation by type-I inducing agents. Furthermore, the temporal trajectories of this expression is consistent with the known mechanisms of such signaling. The expression of IFNB under viral infection was delayed compared to that under stimulation by the TLR ligands poly(I:C) and LPS.

## Conclusions

The bat has been implicated as a major reservoir for viruses of extreme pathogenicity in humans, and suffers substantially less disease when infected by these viruses than humans do. In addition, bat populations in North America are declining rapidly as a result of white-nose syndrome, an emergent disease associated with a fungal pathogen [35]. These facts, and others, suggest that the study of host defense and immunity in these unique creatures would benefit the pursuit of ecological and human health. The major obstacle in this undertaking is the lack of reagents that would make such investigations possible. One way to get this effort underway is to take advantage of the rich store of information contained in existing partial genome sequences. Although the information available in these genome databases requires more careful treatment for its extraction than is required for a complete, assembled genome, we have developed methods that facilitate this task, and have demonstrated their utility.

## Methods

### Ethics Statement

All animals were handled in strict accordance with good animal practice as defined by the relevant national and/or local animal welfare bodies, and all animal work was covered by an IACUC protocol from the Lubee Bat Conservancy (USDA Research Facility #58-R-0131).

### Animals and sample collection

Subjects for this study included 10 male Large Flying Foxes (*Pteropus vampyrus) *Some of the animals were wild caught, and some were laboratory born; all were reproductively mature, ranging in age from 4 to 21 yrs. Animals were housed in captivity at the Lubee Bat Conservancy in Gainesville, Florida, USA. Animals were housed together with conspecifics in an indoor/outdoor circular flight enclosure and were fed a mixture of fruit, vegetables, commercial primate chow, and a vitamin supplement. Water was provided *ad libitum*. The bats were captured manually, anesthesia was mask-induced with 5% isoflurane in oxygen (3 L/min), and the bats were maintained with 2.5% isoflurane. Blood was collected from the left and right brachial veins using 3-ml syringes and 25-gauge needles. Blood samples were transferred to heparanized tubes, maintained and shipped at room temperature (22°C) via overnight courier.

### Cloning and Sequencing

Genomic DNA was prepared from whole unfractionated peripheral blood from *Pteropus vampyrus *bats. Using primers derived from the inferred interferon gene sequences and encoded for restriction enzyme cloning sites, the genes were PCR amplified from the genomic DNA. The forward and reverse primers encoded restriction sites for NheI and XhoI respectively, which allowed cloning of the full length gene into mammalian expression vector pcDNA3.1(+). The PCR program consisted of an initialization step at 94°C for 5 minutes, then 30 cycles of amplification consisting of denaturation at 94°C for 1 minute, annealing at 55°C for 30 seconds, and elongation at 72° for 1 minute. Amplification was followed by a final elongation step at 72°C for 5 minutes and samples held at 4°C. PCR was performed on a P × 2 Thermal Cycler from ThermoElectron Corporation (Waltham, MA).

After purification, the inserted genes were sequenced using vector primers downstream and upstream of the insert site. *Pteropus vampyrus *IFNB, IFNK, IFND, and IFNA sequences have been submitted to Genbank (GU126493, HM63650, HM636501, and HM636502).

#### Quantitative RT-PCR

Whole anticoagulated blood was collected from the brachial vein and layered over lymphocyte separation medium (Lympholyte M, Cedarlane Labs). Cells were collected from the interface, washed, and plated on 24 well flat-bottom tissue culture plates (2 × 10^6 ^cells/well) in a total volume of 250 μl. Cells were treated as indicated with either poly(I:C)(Invivogen), LPS (Sigma), or VSV (Ramsburg lab stocks confirmed endotoxin free using LAL assay). Stimulants were diluted such that the addition of 250 μl stimulant would give a final concentration of 10 μg/ml poly I:C, 1 μg/ml LPS, or an MOI of 5 for VSV. 500 μl of complete medium was added to the unstimulated control wells. Cells were incubated at 37' and 5% CO2 for the indicated times, after which cells were harvested into RNA lysis buffer (Qiagen RNEasy kit) according to the manufacturer's instructions.

Purified RNA was prepared from these whole-cell lysates as described in the protocols accompanying the Qiagen RNeasy kit, Qiashedder, and Qiagen RNeasy Mini kit and Qiagen DNase-Free DNase Set. cDNA synthesis was performed using the Invitrogen two-step qRT-PCR kit according to the manufacturer's protocol. Quantitative real-time PCR was performed in an Eppendorf Mastercycler *ep realplex *thermal cycler using SYBRGreenER qPCR Supermix and primers designed from the inferred gene sequences (additional file 3).

Independent biological replicates were prepared for each treatment and time point. The log_2 _expression level of IFNB and OAS2 relative to the housekeeping gene PPIA was estimated by subtracting the mean Ct value for each gene and treatment/time point combination from the corresponding mean Ct value for PPIA.

### Estimation of Gene Family Size

The likelihood function for the observed set *S *of sequences is a function of the true number, *g*, of genes and the sample size, *n*, by summing over the unobserved number, *c*, of distinct genes sampled.(14)

The pmf on *c *is given by the recursion(15)

with *P_c_*(1 | *g*,1) = 1 and *P_c_*(*c *| *g*,1) = 0 for *c *≠ 1.

*P_S_*(*S *| *c*) is approximated by minimizing the number, *m*, of mutations over the assignments of observed sequences to *c *groups. *P_S _*is then the posterior probability of getting *m *mutations given the observed number of mutations in the IFNB and IFNK datasets.

Note that this technique is independent of the assembly methods described elsewhere in the paper.

### Phylogenetic Inference

The set consisting of type-I interferon amino acid sequences from human (*Homo sapiens*), domestic pig (*Sus scrofa*), duckbill platypus (*Ornithorhynchus anatinus*) and the amino acid sequences corresponding to the Chiropteran genes inferred in this paper was submitted to multiple sequence alignment by CLUSTALW 2.0.10 [36] and ProbCons 1.12 [37]. Maximum likelihood and minimum evolution phylogenies were inferred from each resulting multiple sequence alignment. Maximum likelihood phylogenies were inferred with *proml *found in the PHYLIP 3.68 package [38]. Minimum evolution phylogenies were inferred using *fastme *[39] based on the distance matrix calculated with protdist found in PHYLIP. In both cases, 1000 boot-strap replications were generated using *seqboot *and the *consensus *phylogeny was assembled with consense, both from the PHYLIP package. FigTree 1.2.1 http://tree.bio.ed.ac.uk/software/figtree/ was used for initial visualization and final production of figures of the resulting phylogenetic trees. Alignments of the bat DNA sequences and of all the amino acid sequences are available in additional files 4.

## Authors' contributions

ER and TBK conceived the project and designed the experiments. TBK developed, implemented, and applied the statistical methods. CS, KH, and AH performed the experiments. JR and TBK performed the phylogenetic analysis. AW contributed critical reagents and assisted in interpretation of results. TBK, CS, and ER wrote the manuscript. All authors read and approved the final manuscript.

## Supplementary Material

Additional file 1 **All IFNG.bio**. An alignment of IFNG gene sequences from *Homo sapiens*, *Pterops vampyrus*, and *Myotis lucifugus *as follows: first row: genomic sequence of human IFNG. rows 2, 3: predicted genomic sequence of IFNG in *M. lucifugus *and *P. vampyrus*, respectively. rows 4-7: exons for human IFNG. rows 8-11: predicted exons for *M. lucifugus*. rows 12-15: predicted exons for *P. vampyrus*. row 16: spliced coding region for human. rows 17,18: predicted spliced coding region for *M. lucifugus *and *P. vampyrus*. row 19: amino acid sequence for human interferon gamma. rows 20,21: predicted amino acid sequence for chiropteran interferon gamma. Examination of this. bio file requires the BioEdit program, which is freely available at http://www.mbio.ncsu.edu/BioEdit/bioedit.html. Click here for file

Additional file 2 **Pva IFND clone-assembly comparison.fasta**. This sequence alignment shows a comparison between *Pteropus vampyrus *IFND sequences obtained by 1) assembly from the genome sequencing trace archives, and 2) by direct cloning and sequencing. The cloned sequences include only those found in at least two independent clones. The assemblies include all those with intact ORFs plus three that appear to be pseudogenes. Click here for file

Additional file 3 **Accession numbers and primers**. The Genbank accession numbers of the IFN genes used in the phylogenetic. analysis and the PCR primers used in the *P. vampyrus *gene expression studies. Click here for file

Additional file 4 **Mlu + Pva + Hsa + SscSelectedIntactOrfsAA-aligned.fasta**. The amino acid sequences used in the phylogenetic analysis. Click here for file
