# Supplementary material for: Chiropteran types I and II interferon genes inferred from genome sequencing traces by a statistical gene-family assembler
Source: BMC Genomics. 2010 Jul 21;11:444. doi: 10.1186/1471-2164-11-444 (PMC3091641; doi:10.1186/1471-2164-11-444)
Supplement: Additional file 3 — Accession numbers and primers. The Genbank accession numbers of the IFN genes used in the phylogenetic. analysis and the PCR primers used in the P. vampyrus gene expression studies. [file 1471-2164-11-444-S3.DOCX]

Accession numbers

The Genbank accession numbers of the IFN genes used in the phylogenetic analyses are:

Ornithorhinchus anatinus

BN001099.1, BN001098.1, BN001097.1, BN001096.1, BN001100.1, BN001101.1,

Sus Scrofa

GQ415087.1, NM_001166319.1, GQ415065.1, GQ415066.1, AY687280.1, NM_001164843.1, EU364896.1, GQ415064.1, NM_001166311.1, NM_001164860.1, GQ415062.1, XM_001927328.1, GQ415057.1, NM_001002832.1,

Homo sapiens

M11003.1, BC112302.1, AL353732.14, NM_002169.2, AL162420.13, BC074936.2, NM_002170.3, BC103972.2, NM_021268.2, NM_021068.2, BC114000.1, NM_002175.2, BC146500.1, AB463795.1, V00546.1, NM_176891.4

RT-PCR Primers

The primers used for gene expression studies in P.vampyrus PBMCS are:

IFNB

> Pva IFNB primer F

CTCCCTGCGGAGATTAAACA

> Pva IFNB primer R

CATGCTTTCCCAGGTGAAGT

OAS2

> Pva OAS2 primer F

GTGTTCCCCAGCTCCATTAG

> Pva OAS2 primer R

AACTTCTTCTCCCGCTGACA

PPIA

> Pva PPIA primer F

AGCACTGGGGAGAAAGGATT

> Pva PPIA primer R

GTCTTGGCAGTGCAGATGAA
